# Neuroadaptive Changes Associated with Smoking: Structural and Functional Neural Changes in Nicotine Dependence

**DOI:** 10.3390/brainsci3010159

**Published:** 2013-02-15

**Authors:** Chantal Martin-Soelch

**Affiliations:** 1 Department of Clinical Psychology, University of Fribourg, CH-1700 Fribourg, Switzerland; E-Mail: chantal.martinsoelch@unifr.ch; Tel.: +41-26-300-7687; Fax: +41-26-300-9712; 2 Department of Psychiatry and Psychotherapy, University Hospital Zurich, CH-8091 Zurich, Switzerland

**Keywords:** tobacco, nicotine, brain, reward, human, smokers, dependence, addiction

## Abstract

Tobacco smoking is the most frequent form of substance abuse. We provide a review of the neuroadaptive changes evidenced in human smokers with regard to the current neurobiological models of addiction. Addiction is thought to result from an interplay between positive and negative reinforcement. Positive reinforcing effects of the drugs are mediated by striatal dopamine release, while negative reinforcement involves the relief of withdrawal symptoms and neurobiological stress systems. In addition, drug-related stimuli are attributed with excessive motivational value and are thought to exert a control on the behavior. This mechanism plays a central role in drug maintenance and relapse. Further neuroadaptive changes associated with chronic use of the drug consist of reduced responses to natural rewards and in the activation of an antireward system, related to neurobiological stress systems. Reduced inhibitory cognitive control is believed to support the development and the maintenance of addiction. The findings observed in human nicotine dependence are generally in line with these models. The current state of the research indicates specific neuroadaptive changes associated with nicotine addiction that need to be further elucidated with regard to their role in the treatment of nicotine dependence.

## 1. Introduction

With 1 billion smokers worldwide, tobacco dependence is considered a global public health problem [1]. In the United States, tobacco dependence is one of the leading causes of preventable illness and death [2]. Smoking is associated with cardiovascular disease, cancer and chronic respiratory diseases [3].

Tobacco smokers generally underestimate the addictive effect of smoking and the detrimental consequences of smoking. The addictive property of tobacco is mainly caused by nicotine [4], an alkaloid that binds to neuronal nicotinic acetylcholine receptors [5]. The reinforcing properties of nicotine have been demonstrated with the intravenous self-administration paradigm in rats [6], primates [7] and in human smokers [8]. Nicotine administration increases striatal dopamine (DA) release in experimental animals [6,9,10], a mechanism evidenced in all drugs of abuse that is believed to mediate the reinforcing effects of addictive drugs, because the mesolimbic DA system is crucial in the processing of reward [11,12]. 

Nicotine dependence is characterized by specific withdrawal symptoms, including anxiety, difficulty concentrating, dysphoric or depressed mood, increased appetite or weight gain, insomnia and irritability, frustration or anger [13,14]. Nicotine is often used to relieve these symptoms and nicotine addiction is also characterized by high relapse probability after trying to quit smoking. In a recent report [15], 45% of the smokers reported having attempted to quit in the previous year and stopped for at least one day. Unfortunately, the majority relapsed within 10 days [16,17].

Due to its high prevalence, its detrimental effect on health and the high rates of relapse, it is crucial to get a better understanding of the mechanisms underlying nicotine dependence. Here, we will provide a non-systematical overview of the neuroadaptive changes evidenced in human smokers with regard to the current neurobiological models of substance dependence. 

## 2. Current Neurobiological Models of Substance Dependence

Substance dependence or addiction is nowadays understood in a multifactorial etiological model, which includes psychological, neurobiological, genetic, social and environmental factors [18]. The factors involved in the acquisition of the addiction are differentiated from the factors involved in the maintenance of the dependence. It is postulated that dependence is related to learning processes and that mechanisms of classical and operant conditioning underlie its etiology. 

### 2.1. Vulnerability Factors for Substance Dependence

Genetic factors play an important role in the development of dependence and represent a major vulnerability factor. They can explain up to 50% of the variance observed in individuals with drug or alcohol dependence [19]. It is now hypothesized that there are shared genetic factors for numerous drugs of abuse [20,21,22], contributing to an increased risk for substance dependence in general. In addition, specific genes for each substance of abuse have been identified, representing a vulnerability for the use or abuse of this particular substance [21]. In line with these hypotheses, the research on smoking behavior has focused on genes that may influence the response to nicotine (e.g., nicotine metabolism, nicotinic receptors) and genes that may predispose to addictive behavior in general due to their effects on dopamine (DA) and serotonin neurotransmitters [3]. Furthermore, significant genetic influences on several aspects of smoking behavior have been recently reported [3]. Genetic factors seem to account for approximately 40%–75% of the variation in smoking initiation, 70%–80% of the variation in smoking maintenance, about 50% of the variance in cessation success and 30%–50% of the variance in risk of withdrawal symptoms (see [3] for review).

An accumulation of aversive events or experiences during childhood and adolescence is another vulnerability factor for substance use [23]. The increased levels of cortisol releasing factor (CRF) and cortisol released in reaction to chronic stress have been shown to increase DA transmission and, in the long-term, to induce synaptic changes in the DA system (see [24] for review). Interestingly, neuroimaging studies have evidenced that the DA-D2 receptor density is associated with the subjective feelings elicited by the drugs (see [25] for review). 

Age is a further vulnerability factor for drug use. Adolescence is associated with higher risk for the use of psychoactive substances and for the development of substance dependence. An early onset of drug use increases the probability for drug dependence, as well as the switching from drug abuse to drug dependence [26]. Finally, higher sensation-seeking and impulsivity were evidenced during adolescence [27], two personality factors associated with risk-taking and drug use (see [28] for a review). 

### 2.2. Development and Acquisition of Dependence: The Role of Positive Reinforcement and of the Brain Reward System

#### 2.2.1. Mechanisms of Positive Reinforcement in the Acquisition of Substance Dependence

Substances of abuse are usually taken because of their positive reinforcing effects, *i.e.*, the hedonic feelings induced by the drug. Positive reinforcement is a form of operant conditioning in which a specific behavior is followed by a pleasant or rewarding consequence, which in turn increases the probability of occurrence of this behavior. In the case of substance use, it is postulated that the hedonic feelings elicited by the drug act as a positive reinforcer and increase the probability to use the drug. It is hypothesized that the reinforcing effects of the psychoactive drugs are mediated through the dopamine mesolimbic system, especially through striatal release in the nucleus accumbens, because most substances of abuse, including ethanol, heroin, nicotine, cannabis, amphetamine and cocaine, directly or indirectly increase DA release in this region (see [12,25] for a review of the literature). The mesolimbic DA system and the ventral striatum (including the nucleus accumbens) are also crucial regions of the brain rewarding circuitry, a system of brain regions specifically processing rewarding stimuli [29].

#### 2.2.2. The Hedonic Homeostasis and Mechanisms of Negative Reinforcement in the Acquisition of Substance Dependence

**Figure 1:**
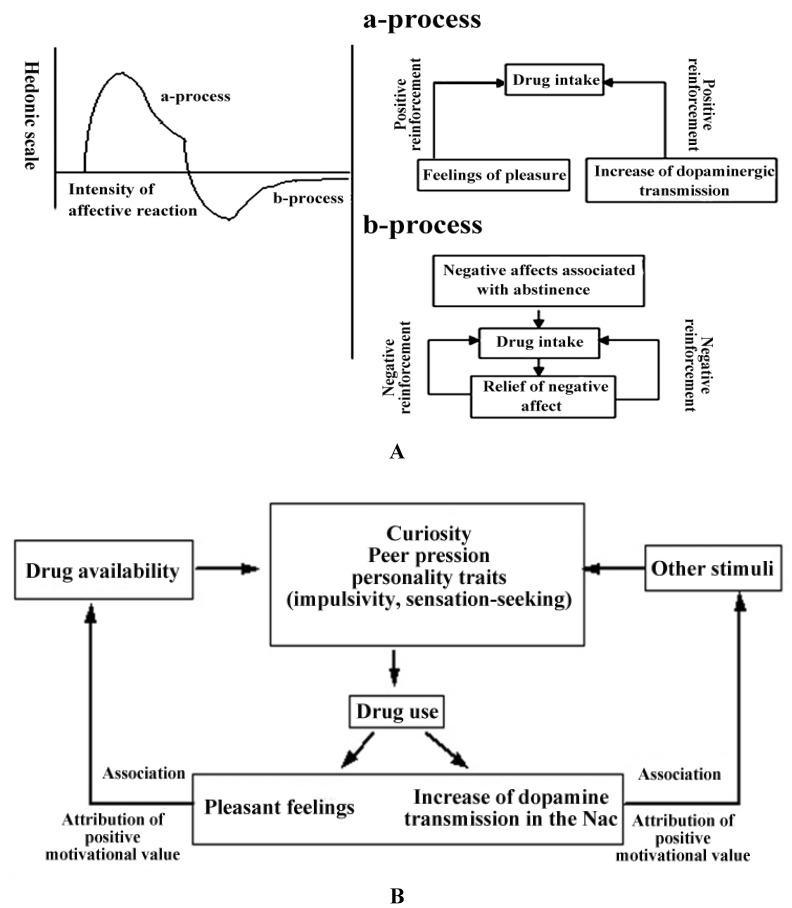
(**A**) Schematic illustration of the model of the hedonic dysfunction postulated by Koob & Le Moal [12]. Adapted with permission from Martin-Soelch [28]. Copyright 2002 Peter Lang. a-Process corresponds to the positive hedonic effects of the substance of abuse; b-process corresponds to the counterregulatory homeostatic reaction of the brain that elicits negative affective states. (**B**) Illustration of the learning processes involved in the acquisition of dependence and in the attribution of incentive motivation to drug related cues. Adapted with permission from Martin-Soelch [28]. Copyright 2002 Peter Lang.

According to the opponent process theory of Solomon [30], every positive affective reaction is followed by a hidden negative process. The hedonic process appears shortly after the presentation of the reinforcer and shows a quick tolerance. In contrast, the negative process begins when the hedonic effects begin to fade; it decreases slowly and is reinforced with repeated presentation of the reinforcer. In the context of drug dependence, it means that the body’s own hedonistic homeostasis is disturbed by overstimulation associated with the use of the drug (see Figure 1A). The brain reacts with counterregulatory homeostatic mechanisms that are associated with negative affective states. These dysphoric emotional states are in turn seen as motivation factors for the maintenance of drug dependence [31]. It is further hypothesized that the positive reinforcing effects of the substances of abuse decrease with repeated use, while the mechanisms of negative reinforcement (*i.e.*, the use of the drug to relieve the dysphoric emotional states) get stronger and represent a major motivational determinant of the maintenance of substance dependence ([12]).

The transition from substance abuse to substance dependence is related to the counterregulatory homeostatic mechanisms (see Figure 1A), which elicit negative affective states and withdrawal symptoms. These processes are thought to be associated with an activation of neurobiological stress systems, including CRF and norepinephrine transmission [32]. The substance of abuse will be used again in order to relieve these negative symptoms. The behavior is therefore reinforced by the withdrawal of an unpleasant event, which in turn corresponds to the mechanism of negative reinforcement [33]. In this framework, substance dependence results from an interplay between positive and negative reinforcement. 

#### 2.2.3. The Conditioning of Drug-Related Cues

A further important factor in the development of substance dependence is the conditioning of drug-related stimuli with the reinforcing effect of the substance. The DA release elicited by the substances of abuse facilitates the learning of incentive stimuli predicting the availability of the drug, the so-called drug cues, through associative learning [11,34] (see Figure 1B). In addition, the increase in DA release elicited by the drug of abuse is not subject to habituation, as opposed to the DA release in response to natural reinforcers [11], which in turn reinforces the association between drug-related reward and drug-related cues in an abnormal way. Drug-related cues are attributed with excessive motivation value and can exert a control on the behavior. 

#### 2.2.4. The Role of the Dorsal Striatum in the Development of Addiction Habits

From a neurobiological point of view, it has been hypothesized that the transition from voluntary drug use to habitual response compulsive drug use is associated with a shift in the locus of control from the ventral to the dorsal striatum [35]; and the dorsal striatum is also known to be involved in the development of motor habits [25]. This shift occurs through the regulation of the dorsal striatum by the ventral striatum over ‘spiraling’ connections with the midbrain [35,36]. This transition from the ventral striatum to the dorsal striatum could lead to the development of addictive habits that could in turn explain the loss of control over drug-seeking behavior. 

Therefore, the high change resistance of smoking behavior could be explained by the fact that drug-taking and drug-seeking behaviors have become habitual and automatized processes [37]. Some findings in human smokers, for instance, support this hypothesis and show that smoking behavior becomes automatized in frequent smokers [38,39]. Although the hypothesis of a role of the dorsal striatum in the development of drug habits is mostly based on animal research, some recent findings in human have evidenced a role of the dorsal striatum in addiction. PET (Positron Emission Tomography) studies showed that drug-related cues could increase DA release in the dorsal striatum in cocaine users and that these increases were associated with cocaine craving ([40,41]. An fMRI-study of alcohol cue reactivity in heavy and light social drinkers evidenced higher cue-induced activation of the ventral striatum in the light compared to heavy drinkers, while higher dorsal striatal activation was found in heavy drinkers [42]. 

### 2.3. Maintenance of Dependence and Relapse: The Role of Drug Cues, Addictive Memory and of Neuroadaptation

The chronic use of the substance is associated with long-term neuroadaptive changes, consisting in (1) an excessive incentive value of drug cues and (2) the creation of a drug-related memory [18]. After only a few administrations of the substance, a long-lasting hyperactivity of the mesolimbic DA system develops that leads to a higher reactivity of this system [34]. The association between environmental cues and the activation of the mesolimbic DA system leads to the creation of an implicit memory, which could be coded at the neural level in the form of memory engrams [43,44]. This learning process involves the amygdala, the hippocampus, the frontal cortex and the inferior parietal cortex and creates an individual addictive memory. These brain regions also influence the cortico-striatal loop, which plays a major role in the development and maintenance of substance dependence [44]. This individual addictive memory can be reactivated even after a long abstinence time, because of the neurochemical sensitization and it is associated with an augmented attention towards drug-related cues and drug craving. 

Further neuroadaptive changes consist, on one hand, in the activation of an antireward system, which induces long-lasting negative states; and, on the other hand, in the reduction of the rewarding effects of natural rewards [33] The antireward systems are related to the brain stress systems, such as CRF, norepinephrine and dynorphin, that produce aversive or stress-like states [45]. The role of the antireward system is to maintain the hedonic homeostasis, as hypothesized in the motivational opponent process theory ([30], see Figure 1A). Koob [45] postulates that the combination of the diminished function in the reward system and the recruitment of the antireward system constitutes the neurobiological basis for motivational withdrawal, as well as a strong source of negative reinforcement that is implicated in compulsive drug-seeking behavior and relapse. The negative emotional states that are observed in human addicts during the withdrawal/negative affect stage can also be elicited in animals during withdrawal from all major drugs of abuse and are reflected by increases in anxiety-like behavior, dysphoric-like responses and reward thresholds [45].

**Figure 2:**
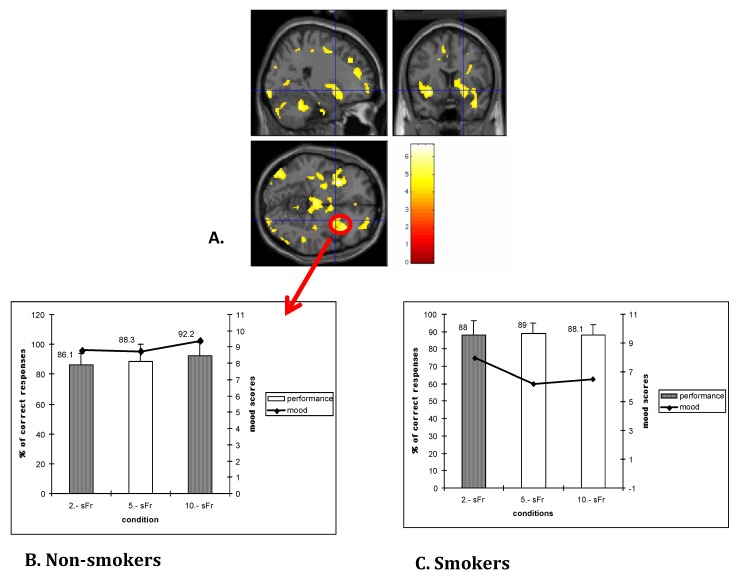
(**A**) Increased activation in the right striatum (putamen) in non-smokers in response to monetary rewards; in smokers, no striatal activation was found in response to monetary reward [47]. (**B**) The striatal activation was correlated with higher mood ratings evidenced in non-smokers in relation to increasing monetary rewards (CHF 2, CHF 5, CHF 10). (**C**) In smokers no mood increase was observed in association with increases of monetary rewards [48].

The hypothesis of a reduced function of the brain reward system is supported by previous PET studies by our group showing a reduced activation of the striatum in response to monetary rewards in heroin-dependent subjects and in smokers [46,47,48] (see Figure 2). In addition, smokers, as well as cannabis users, showed a reduced effect of monetary rewards on mood [48,49]. In addition, the persistent negative emotions, the stress states, as well as the presence of drug-related cues, increase the risk for relapse in several groups of dependent-subjects, including abstinent smokers (see [24] for review). 

### 2.4. Cognitive Control Dysregulation and Deficits in Executive Functions

#### 2.4.1. Decision-Making Deficits and the Ventromedial Prefrontal Cortex

In the previous sections, the emphasis was on learning and motivational mechanisms involved in the development and maintenance of dependence. A limitation of these models is that they almost completely disregard cognitive processes, especially decision-making processes. For instance, it is not enough that a substance of abuse is available—the individual still has to decide to use it; and not all the individuals having tried a substance of abuse become dependent on this substance; there is always a conscious decision to use the substance again. Bechara [50] postulates that substance-dependent individuals have deficits in decision-making. He showed that substance-dependent subjects evidenced similar deficits as patients with lesions in the ventromedial prefrontal cortex (vmPFC) in a decision-making task (the Iowa Gambling Task) [52]. These patients made decisions associated with immediate high wins, even if these decisions were associated with higher losses in the long-term [52]. Healthy subjects, on the contrary, would prefer medium wins that are associated with medium losses and have a positive balance at the end of the game. The hypothesis of a disturbed decision-making behavior is supported by several studies in substance-dependent subjects (see [53] for review). However, it remains unclear whether these deficits are a cause or a consequence of substance-dependence. Another limitation of this hypothesis is that a detrimental relationship between some aspects of impulsivity and decision-making was also evidenced in healthy subjects [54], suggesting that the deficits in decision making observed in substance-dependent individuals could be rather associated with impulsivity than with the consequences of substance use.

#### 2.4.2. Impaired Insights and the Insula

A further cognitive deficit observed in substance dependence is the lack of insight in the disease [55]. For instance, a study of heavy drinkers reports that only few of the examined subjects perceive their behavior as problematic, even after they were confronted with the negative consequences of their drinking behavior [56]. This lack of insight could be associated with dysfunction of the insula and anterior cingulate gyrus (ACC) [55]. The insula codes the interoceptive states of the body, integrates emotional salient information and is activated by subjective physical and emotional feelings [57]. Insula and the anterior cingulate cortex can be seen as two complementary regions that are jointly activated in most human emotions and behaviors [57]. Reduced ACC activation was observed in cocaine, heroin, alcohol and cannabis users during selective attention and inhibitory control [58]. A further study showed that reduced activation in ACC and amygdala could predict diminished error perception in a performance test in cannabis users [59]. The insula is also involved in the urge to take drugs. Naqvi *et al.* [60] reported that smokers with insula lesions were >100 times more likely than smokers with brain damage not involving the insula to undergo a disruption of smoking dependence, characterized by the ability to quit smoking easily, without relapse and without a persistence of the urge to smoke. 

#### 2.4.3. Dysfunctional Inhibitory Systems

An attempt to integrate learning, motivation and cognitive neural models of dependence was made by Bechara [50], who postulated that substance-dependence could be regulated by an impulsive system and a dysfunctional inhibitory system. The impulsive system would principally involve the amygdala and the striatal DA reaction to drug-related cues, while the inhibitory regions would involve the vmPFC and dorsolateral prefrontal cortex (DLPFC), two regions implicated in the cognitive deficits observed in substance-dependent subjects. These inhibitory mechanisms are involved in the regulation of internal motivational states and can suppress reflexes and conditioned reactions, allowing slower cognitive processes to join in order to regulate the behavior. In this context, it is postulated that chronic drug use is associated with a reduction of these inhibitory processes and that the already strong association between drug-related cues and drug reward gets an even stronger control over the behavior. 

## 3. Findings in Human Smokers

We will here provide a short overview of the structural and functional neural changes observed in human smokers with regard to the different mechanisms involved in the current models of drug dependence that were discussed in the previous section. 

### 3.1. Changes in Brain Regions and Neurotransmitter Systems Associated with the Brain Reward Circuitry

According to the hypothesis that the positive reinforcing properties of the substances of abuse are mediated through DA transmission in regions associated with the cerebral reward system, we will present here neurochemical or structural changes observed in smokers in striatal regions and in the DA system. Studies related to reduced neural reactions to natural rewards are presented in the next section.

#### 3.1.1. Neurochemical Changes in Smokers

Several [61,62,63], but not all [64,65], neuroimaging studies of smokers could show that smoking is associated with increases in DA release in the ventral striatum. DA release was shown to be associated with the mood changes elicited by nicotine [66] and to be modulated by genes associated with low resting dopamine tone [61]. There is also some evidence for changes in endogenous opioid transmission in response to smoking [67] that seems to be also influenced by genetic factors [1]. Neuroreceptor studies of DA generally point to a reduced DA function in smokers. Fehr *et al*. [68] showed that nicotine-dependent men exhibited lower putamen D2/D3 dopamine-receptor availability than non-smokers. This effect was, however, evidenced only in males and not in female smokers [69]. The results related to dopamine transporter (DAT) availability are more consistent and suggest a reduced DAT availability in smokers [70,71,72]. 

#### 3.1.2. Structural Changes and Functional Connectivity

Volumetry studies investigating structural changes in the striatum failed to find clear structural changes in the striatum [73,74], but evidenced the indirect effect of lifetime cigarette smoking on the size of specific striatal regions, including the nucleus accumbens and the putamen [73]. 

Studies investigating measures of functional connectivity, *i.e.*, analyses looking at statistical associations between the activation in different brain regions, could evidence some differences between smokers and non-smokers in regions associated with the brain reward circuitry. A recent study showed that smokers had greater coupling *versus* non-smokers between left fronto-parietal and medial prefrontal cortex networks; and smokers with the greatest medial prefrontal–left fronto-parietal coupling had the most dorsal striatal smoking cue reactivity, as measured during an fMRI smoking cue reactivity task [75]. Hong *et al*. [76] identified two bilateral dorsal anterior cingulate gyrus to ventral striatal circuits whose connectivity strengths were inversely proportional to an individual’s level of nicotine dependence, as measured by Fagerström scores, a widely used test to assess the severity of nicotine dependence [77]. A subsequent study by this group [78] demonstrated that a gene variant of the α5 subunit of nicotinic acetylcholine receptors, one of the most replicated genetic marker of smoking [79], was associated with decreased resting state functional connectivity in a dorsal ACC-ventral striatum/extended amygdala circuit in smokers expressing the risk allele. This circuit was anatomically consistent with that previously shown to predict dependence severity using the phenotypic Fagerström index [80].

Interestingly, both the neurochemical, as well as the functional changes observed in smokers in regions associated with DA and the cerebral reward system, seem to be influenced by specific genes, suggesting a genetic predisposition of the neural mechanisms involved in the acquisition of dependence in smokers.

### 3.2. Changes in Brain Regions and Neurotransmitter Systems Associated with Smoking-Associated Cues and the “Anti-Reward” System

Most studies investigating the neuroadaptive changes associated with the maintenance of nicotine dependence have investigated the reaction to natural reward or to smoking cues during abstinence. Drug-related cues are believed to trigger compulsive drug seeking. The craving elicited by smoking-related cues are often reported by smokers as the precipitating cause of relapse [81]. 

The hypothesis of reduced reactions to natural reward was mostly confirmed in smokers. One study showed reduced cue reactivity, pleasure expectancies and responsiveness to financial incentives in abstinent smokers [82], while two recent fMRI studies evidenced a reduced striatal activation in response to natural or monetary rewards in (not-abstinent) smokers [83,84], confirming our previous results obtained with the PET-method [47,48] (see Figure 2).

Further neuroimaging studies of smokers reported increased activation in regions associated with the mesolimbic DA system during the presentation of cigarette cues compared with neutral cues [85,86,87] that was potentiated during smoking abstinence [88]. This excessive effect of smoking cues on neural activation was shown to be modulated by genes related to the dopamine transporter [89] and to be reduced by administration of varenicline, a smoking cessation medication that functions as a partial agonist of nicotinic receptors [90]. An attentional bias toward smoking cues was also identified in abstinent smokers, which was related to activation in DLPFC, putamen, posterior cingulate cortex and primary motor cortex [91] and was shown to overshadow the value of neutral cues predicting natural reward [92]. Finally, the neural response to smoking cues compared to the neural activation to pleasant stimuli was shown to be predictive of abstinence [93]. 

Finally, several studies supported the hypothesis that the dorsal striatum is involved in the development of addictive habits in smokers and reacts to smoking cues. Yalachkov *et al.* [37] showed higher fMRI activations in smokers than nonsmokers when viewing smoking-related compared to control images in the dorsal striatum, but also in regions implicated in the encoding of action knowledge and tool use skills, such as the premotor cortex, the superior parietal lobule and the right lateral cerebellum. Smolka *et al.* [94] reported correlations between severity of dependence and brain activity in regions involved in motor preparation in response to smoking cues in a group of smokers with different severity levels of smoking dependence. Finally, Janes *et al*. [95] observed greater fMRI activity during extended abstinence compared to before smoking cessation in the dorsal striatum in response to smoking cues. A further study by this group showed that the neural reaction to smoking cues before entering a smoking cessation program predicted relapse, especially the activation in the insula and in the dorsal striatum [96].

Taken together, these findings are consistent with the excessive incentive value of drug-related cues playing a role in the maintenance of nicotine dependence and with the implication of brain regions involved in the development of motor habits in response to smoking cues in smokers.

### 3.3. Changes Associated with Cognitive Control Systems

Further neuroadaptive changes postulated in dependence models are related to the reduced influence of prefrontal inhibition mechanisms and cognitive deficits, including impaired decision-making and insight in the disease. A specificity of nicotine dependence is that nicotine has enhancing properties on cognitive performance [97], suggesting a direct effect of nicotine on brain regions involved in cognitive processes. Therefore, it is not surprising that studies of resting state functional connectivity could evidence seven cingulate-neocortical pathways that demonstrated enhanced connectivity strength in the presence of an acute nicotine patch in smokers [75]. These circuits were consistent with those implicated in the performance-enhancing properties of nicotine [97]. 

In line with the hypothesis of reduced prefrontal control mechanisms, a functional neuroimaging study evidenced reduced prefrontal cortical activity in current smokers compared to ex-smokers in response to smoking cues and to a motor response inhibition task [98]. A study of twin pairs discordant for regular cigarette smoking showed that regular smoking had robust effects on regions associated with cognitive control, but modest effects on regions associated with reward processing regions during a reward guessing task [99]. A further study provides the first indication for an association between smoking withdrawal and the greater recruitment of insular, frontal and parietal cortical areas during a gambling task [100]. Finally, volumetry studies evidenced reduced cortical grey matter volumes in the frontal and temporal lobes of smokers [74,101].

In summary, the findings related to the neural cognitive control circuits not only provide evidence of connectivity and structural changes in these regions, but also indicate a dysfunction in these circuits during reward processing and decision-making.

## 4. Conclusion

The findings observed in human nicotine dependence are in line with the current models of dependence. An increase of DA release in the striatum in response to smoking was observed in smokers, supporting the positive reinforcing effects of nicotine. A reduced DA function was also generally observed in smokers as it has been in other types of dependence (see [25] for review). An important step towards compulsive drug use consists in the conditioning of drug-related stimuli that are associated with excessive motivational value and are thought to exert control on the behavior through the ventral striatum. A further step is the involvement of the dorsal striatum in response to these cues that contributes to the development of addictive habits, which in turn become automatized. The investigation of the effect of smoking cues has been intensively studied, and the majority of these studies confirm that smoking cues elicit increases in the neural activation in regions related to the mesolimbic reward system and in regions associated with the development of motor habits. Furthermore, the neural reactions to smoking cues have been shown to be potentiated during abstinence and to be predictive of relapse in smokers. The neuroadaptive changes associated with chronic use of the drug that are thought to maintain the drug taking behavior consist, among others, in reduced neural responses to natural rewards. Studies in smokers could evidence reduced reaction to natural and financial rewards during abstinence, as well as structural and functional changes in brain regions associated with the cerebral reward system (for instance in striatal regions). Finally, a reduced inhibitory cognitive control associated with impaired cognitive functions is believed to support the development and the maintenance of dependence. In smokers, dysfunctional activity in cortical regions associated with cognitive control was also observed during cognitive tasks and during reward processing. Interestingly many findings related to the neuroadaptive changes associated with smoking point to the influence of genetic factors on these changes.

The current state of the research, therefore, indicates specific neuroadaptive changes associated with nicotine addiction that need to be further elucidated with regard to their role in the treatment of nicotine dependence.
